# Deep brain stimulation of the nucleus basalis of Meynert modulates hippocampal–frontoparietal networks in patients with advanced Alzheimer’s disease

**DOI:** 10.1186/s40035-022-00327-9

**Published:** 2022-12-05

**Authors:** Yin Jiang, Tian-Shuo Yuan, Ying-Chuan Chen, Peng Guo, Teng-Hong Lian, Yu-Ye Liu, Wei Liu, Yu-Tong Bai, Quan Zhang, Wei Zhang, Jian-Guo Zhang

**Affiliations:** 1 Department of Functional Neurosurgery, Beijing Neurosurgical Institute, Capital Medical University, Beijing, 100070 China; 2 Department of Functional Neurosurgery, Beijing Tiantan Hospital, Capital Medical University, Beijing, 100070 China; 3 Center for Cognitive Neurology, Department of Neurology, Beijing Tiantan Hospital, Capital Medical University, Beijing, 100070 China; 4 Beijing Key Laboratory of Neurostimulation, Beijing, 100070 China

**Keywords:** Alzheimer’s disease, Deep brain stimulation, Nucleus basalis of Meynert, Hippocampal, Frontoparietal, Functional connectivity

## Abstract

**Background:**

Deep brain stimulation (DBS) of the nucleus basalis of Meynert (NBM) has shown potential for the treatment of mild-to-moderate Alzheimer’s disease (AD). However, there is little evidence of whether NBM-DBS can improve cognitive functioning in patients with advanced AD. In addition, the mechanisms underlying the modulation of brain networks remain unclear. This study was aimed to assess the cognitive function and the resting-state connectivity following NBM-DBS in patients with advanced AD.

**Methods:**

Eight patients with advanced AD underwent bilateral NBM-DBS and were followed up for 12 months. Clinical outcomes were assessed by neuropsychological examinations using the Mini-Mental State Examination (MMSE) and Alzheimer’s Disease Assessment Scale. Resting-state functional magnetic resonance imaging and positron emission tomography data were also collected.

**Results:**

The cognitive functioning of AD patients did not change from baseline to the 12-month follow-up. Interestingly, the MMSE score indicated clinical efficacy at 1 month of follow-up. At this time point, the connectivity between the hippocampal network and frontoparietal network tended to increase in the DBS-on state compared to the DBS-off state. Additionally, the increased functional connectivity between the parahippocampal gyrus (PHG) and the parietal cortex was associated with cognitive improvement. Further dynamic functional network analysis showed that NBM-DBS increased the proportion of the PHG-related connections, which was related to improved cognitive performance.

**Conclusion:**

The results indicated that NBM-DBS improves short-term cognitive performance in patients with advanced AD, which may be related to the modulation of multi-network connectivity patterns, and the hippocampus plays an important role within these networks.

***Trial registration*:**

ChiCTR, ChiCTR1900022324. Registered 5 April 2019—Prospective registration. https://www.chictr.org.cn/showproj.aspx?proj=37712

**Supplementary Information:**

The online version contains supplementary material available at 10.1186/s40035-022-00327-9.

## Background

Alzheimer's disease (AD), a neurodegenerative disease characterized by progressive episodic memory impairment, is the most common form of dementia and a major burden to family members and healthcare services [1]. Although cholinesterase inhibitors and glutamine *N*-methyl-*D*-aspartate receptor antagonists are widely used for treatment of AD, no drugs are currently available to reverse or even alleviate neurodegeneration [2–4]. Recent preclinical studies and clinical trials have investigated deep brain stimulation (DBS) as a potential treatment for dementia-related disorders [5–9]. The nucleus basalis of Meynert (NBM), a cholinergic nucleus in the basal forebrain that provides extensive projections to all cortical areas, is a common target of DBS for treatment of AD [10].

As first reported in 1985, DBS of the NBM (NBM-DBS) in a 71-year-old AD patient over a period of 9 months ameliorated the reduction of cortical glucose metabolism in the stimulated hemisphere, although cognitive function remained unchanged [1]. Thirty years later, in 2015, Kuhn et al. [12] conducted a phase 1 study to evaluate the safety and efficacy of NBM-DBS in six patients with mild-to-moderate AD and found that long-term DBS was well tolerated, and clinical stability or even improvement was found in 4 of the 6 patients after 12 months. Later, a case report of two patients published in 2015 suggested that stabilization of cognitive decline was more effective in younger, less affected patients [2]. Recently, our group reported improved clinical symptoms in an 80-year-old patient with severe AD after 10 weeks of NBM-DBS [3]. However, there is still little evidence of whether NBM-DBS can improve cognitive function in patients with advanced AD.

To date, most studies exploring the mechanism underlying the neuromodulation by NBM-DBS have been conducted in animal models [4]. These studies found that NBM-DBS increases the release of acetylcholine, enhances cerebral blood flow, induces release of several neuroprotective factors, and facilitates plasticity of the cortical and subcortical receptive fields. In human studies, NBM-DBS increases cerebral metabolism, as detected by positron emission tomography (PET), mainly in the medial temporal lobe [5]. Although previous studies have mostly focused on local brain areas, magnetic resonance imaging (MRI) performed in the resting state has revealed atypical patterns of functional connectivity in large-scale brain networks in AD patients [6–8]. Because NBM induces widespread efferent projections to the entire neocortex and altered NBM-cortical functional connectivity is closely related to cognitive impairment [9], it is reasonable to speculate that NBM-DBS could also induce modulation of brain network patterns. Thus, the aim of the present study was to investigate whether NBM-DBS could modulate cognitive function and brain network connectivity in patients with advanced AD and to explore the underlying mechanisms.

## Materials and methods

### Study approval and patient consent

The study protocol was approved by the Ethics Committee of Beijing Tiantan Hospital (Beijing, China) (Approval No. KY 2018-051-02) and conducted in accordance with the ethical principles for medical research involving human subjects as defined in the Declaration of Helsinki. Written informed consent was obtained from all patients and/or their relatives.

### Participants

The study cohort included five women and three men aged 59–78 years who met the diagnostic criteria for AD of the National Institute of Neurological and Communicative Disorders and Stroke and the Alzheimer's Disease and Related Disorders Association [10]. All participants in this study were diagnosed with moderate-to-severe dementia (Mini-Mental State Examination [MMSE] score, 2–16) [11, 12]. Seven of the study participants were taking a consistent dosage of an acetylcholinesterase medication for at least 3 months prior to study initiation, while one (subject 4) reported no medication use for at least 3 months. The exclusion criteria included mild AD (MMSE score > 20), suicidal tendencies, previous intracranial intervention, and contraindication to anesthesia, MRI, or PET.

### Surgery and stimulation

**Fig. 1:**
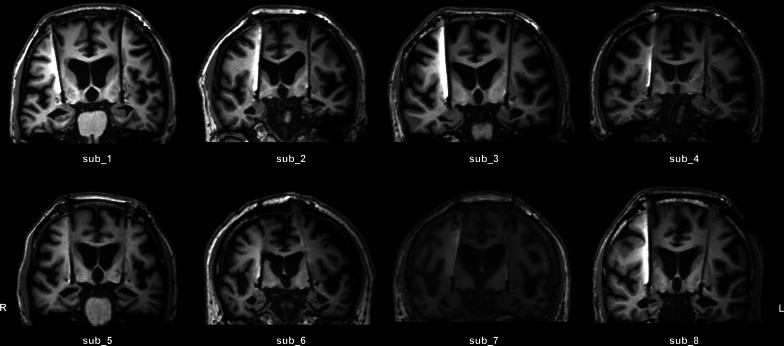
Post-operative MRI showed the NBM lead location for each subject (*n* = 8)

As previously reported, DBS electrodes were placed in the bilateral NBM [3, 13]. Briefly, DBS electrodes (L301C; Beijing PINS Medical Co., Ltd., Beijing, China) were implanted into the T2-weighted MRI-identified NBM (Ch4p) target using a Leksell stereotactic system (Elekta Instrument AB, Stockholm, Sweden) under local anesthesia. In our study, subjects were targeted at an average of 7 mm posterior to the anterior commissure, 7 mm inferior to the ACPC line, and 25 mm lateral. Specific coordinates for each subject are given in Table 1. A neurostimulator (G106R; Beijing PINS Medical Co., Ltd.) was also connected to the leads (model E202C; Beijing PINS Medical Co., Ltd.). This DBS system is reportedly safe for long-duration 3 T MRI [14–16]. Postoperative MRI was performed to confirm the location of the leads and identify signs of cerebral hemorrhage after surgery (Fig. 1). Stimulation began 1 month after surgery. The stimulation parameters were determined based on previous clinical studies [5, 17]. Continuous stimulation was delivered at 20 Hz and 2.0–3.0 V with a pulse width of 90 μs.

**Table 1:** Demographic data, medication, target coordinates, and stimulation parameters

Patient	Age/Sex	Baseline		Medication (daily dose)	Target coordinates^a^ (x,y,z)	Contacts^b^	Stimulation setting
		MMSE	ADAS-Cog				
sub_1	61/F	5	64	Donepezil (5 mg)	− 28.5, − 5.0, − 7.4	C + 2 − /C + 6 −	20 Hz, 90 μs, 2.0 V
					26.9, − 7.3, − 7.6		
sub_2	67/M	9	40	Donepezil (10 mg)	− 27.2, − 4.7, − 7.0	C + 2 − /C + 6 −	20 Hz, 90 μs, 2.5 V
				Memantine (20 mg)	26.0, − 5.2, − 9.0		
				Aniracetam (0.6 g)			
sub_3	66/M	8	37	Donepezil (10 mg)	− 24.6, − 7.1, − 8.7	C + 2 − /C + 6 −	20 Hz, 90 μs, 3.0 V
				Memantine (20 mg)	25.6, − 6.2, − 7.7		
sub_4	78/M	16	19	/	− 26.3, − 6.1, − 10.1	C + 2 − /C + 6 −	20 Hz, 90 μs, 2.5 V
					25.9, − 5.5, − 9.5		
sub_5	67/F	7	62	Donepezil (5 mg)	− 23.8, − 9.2, − 5.2	C + 2 − /C + 6 −	20 Hz, 90 μs, 3.0 V
				Memantine (2.5 mg)	22.5, − 10.1, − 6.4		
sub_6	70/M	4	58	Donepezil (10 mg)	− 25.2, − 6.0, − 6.5	C + 2 − /C + 6 −	20 Hz, 90 μs, 2.5 V
					24.1, − 6.7, − 5.5		
sub_7	59/F	4	63	Donepezil (10 mg)	− 24.8, − 6.3, − 7.6	C + 2 − /C + 6 −	20 Hz, 90 μs, 2.5 V
				Huperzine A (0.1 mg)	26.1, − 6.1, − 5.4		
				Oxiracetam (0.4 g)			
sub_8	65/M	2	60	Donepezil (10 mg)	− 20.7, − 7.8, − 7.4	C + 2 − /C + 6 −	20 Hz, 90 μs, 2.5 V
				Memantine (5 mg)	25.1, − 7.2, − 7.8		

^a^Left and right hemisphere target coordinates. ^b^Active monopolar contacts for the open stimulation phase

### Clinical evaluation

Clinical assessments at different time points were performed by experienced neurologists. Baseline assessments were conducted 1 week before surgery. Postoperative follow-up assessments were conducted at 1, 3, 6, and 12 months after surgery. Clinical assessments included MMSE and the cognitive subscale of the Alzheimer’s Disease Assessment Scale (ADAS-Cog). It should be noted that because stimulation was performed 1 month after surgery, assessment at 1 month after surgery was performed at 3–5 days after stimulation.

### MRI data acquisition

Structural and functional MRI (fMRI) data were acquired with a 3.0 T MRI scanner equipped with a 32-channel head coil (Philips Healthcare, Andover, MA). Structural images were acquired using a magnetization-prepared rapid acquisition gradient echo sequence: repetition time (TR) = 6.57 ms, echo time (TE) = 3.02 ms, flip angle (FA) = 8°, voxel size = 1 mm × 1 mm × 1 mm, field of view (FOV) = 240 × 240 mm^2^, and matrix size = 256 × 256 mm^2^. Blood oxygen level-dependent (BOLD) images were obtained using the following echo-planar imaging sequence: TR = 2000 ms, TE = 30 ms, FA = 90°, FOV = 240 × 240 mm^2^, acquisition matrix = 64 × 64, number of slices = 40, voxel size = 3 mm × 3 mm × 4 mm, and measurement = 180.

Scans by structural MRI and resting fMRI were acquired at 1, 6, and 12 months after surgery. The fMRI runs consist of one on the DBS-off state and one on the DBS-on state. The order of scans in the DBS-off and -on states was balanced across patients. The DBS-off state was maintained for at least 2 h before scanning. The patients were instructed to relax with their eyes closed and not to sleep during the scan. Because the patients had moderate-to-severe cognitive impairment, each was accompanied by a caregiver in the scanning room during the scans. If the patient could not cooperate, the data collected at that time point were abandoned.

### fMRI data preprocessing

Statistical Parametric Mapping (SPM12) software (Wellcome Department of Cognitive Neurology, London, UK) and MATLAB software (MathWorks, Natick, MA) were used for statistical analysis of the data, which were preprocessed as reported previously [18]. In this study, all DBS apparatus-induced magnetic susceptibility artifacts were in the left brain and mainly located in the partial left inferior parietal lobe, temporal lobe, occipital lobe, and cerebellum. Enantiomorphic normalization methods (mirror image normalization) were used to fill the artifacts to enhance the accuracy of normalization [19, 20]. The artifact-filled images were then preprocessed according to a standard pipeline; the first 10 volumes of each session were removed and then all volumes were realigned and spatially normalized to a standard template and spatially smoothed using a Gaussian filter (6-mm full width at half maximum).

### Independent component analysis (ICA)

ICA was conducted using the Group ICA of fMRI Toolbox (GIFT V4.0; http://icatb.sourceforge.net/). The number of independent components was set to 30 based on the estimating results of the minimum description length criteria. After back reconstruction, the spatial components were converted to z-maps for each individual scan. The z-maps of each component were then submitted to a one-sample *t*-test and spatially sorted to the anatomy templates of resting-state networks in the toolbox. Six components were selected for further analysis, including the salience network (SN), hippocampal network (HIPP), default mode network (DMN, which consist of frontal component and parietal component), and frontoparietal network (FPN, which consists of left and right FPN components) (Additional file 1: Fig. S1). The functional connectivity among these four networks was further compared between the DBS-off and -on states using the MANCOVAN toolbox.

### Seed-based functional network analysis

Region-to-region functional connectivity was detected by seed-based connectivity analyses. The preprocessed data were linear-detrended and bandpass-filtered (0.01–0.08 Hz), and nuisance covariates (i.e., white matter, cerebrospinal fluid signals, and head motion parameters) were regressed out. In total, 14 seeds in the above-mentioned four networks of interest were selected based on high *t*-value clusters in the *t*-maps generated by the one sample *t*-test within each component, and 6-mm spheres were drawn using the MarsBar SPM toolbox. The 14 seeds included the dorsal anterior cingulate cortex (dACC) and the left and right insula (INS) in the SN; left and right parahippocampal gyri (PHG) in the HIPP; medial prefrontal cortex (mPFC), posterior cingulate cortex, and right inferior parietal lobule in the DMN; and left and right anterior lateral prefrontal cortex (alPFC), left and right frontal cortex, and left and right parietal cortex (PC) in the FPN (Additional file 1: Fig. S2). For each seed, the time-course of BOLD signal was extracted from each scan. Correlations among these seeds in the DBS-off and -on states were calculated and compared. Then, the connectivity values were correlated to the clinical scores.

Granger causality analysis (GCA) of the changed connections of functional connectivity was conducted to identify the causal influence flows changed by NBM-DBS. ROI-wise GCA was implemented on REST software (http://www.restfmri.net/forum/REST-GCA) using multivariate coefficients. For each scan, there was a coefficient between every two seed regions. The coefficient value was further submitted to a group-specific one-sample test and compared between the DBS-off and DBS-on states.

### Dynamic functional connectivity analysis

Temporal variations of functional connectivity among the 14 seeds were identified using the DynamicBC Toolbox [21]. Dynamic functional connectivity was investigated with the sliding window approach. In line with previous studies [22, 23], the resting-state data were divided into windows of 20 repetition times (40 s), in steps of one repetition time. Thus, each dataset generated a series of time-varying matrices that captured dynamic changes to functional connectivity among the 14 seeds. Afterward, all functional connectivity matrices were transformed to z-scores using Fisher’s z-transformation.

The *k*-means algorithm was applied to a series of time-varying matrices based on the Manhattan distance [24]. The optimal number of clusters was 3 (*k* = 3). Changes of the state of functional connectivity were then captured. The “fractional window”, as the number of total windows belonging to one state, was compared between the DBS-off and -on states and correlated with the clinical scores.

### PET image acquisition and analysis

Patients underwent [^18^F]-2-deoxy-2-fluoro-*D*-glucose PET (FDG-PET) at pre-surgery and at 12 months of follow-up at the DBS-on state. After injection with a radiotracer (5 mCi ± 10%) and a 30-min interval to allow for uptake, a CT transmission scan was acquired followed by a static emission scan beginning 40 min post-injection (acquisition, 20 min; quantification, last 10 min). The standardized uptake value (SUV) was calculated on a voxel-wise basis using the formula SUV = (radioactivity concentration in each voxel)/(decay corrected injected dose/body weight).

Pre-processing and statistical analyses of the PET SUV images were performed with the SPM12 software as described in our previous study [25]. Briefly, images were spatially normalized onto the Montreal Neurological Institute atlas and smoothed using a Gaussian filter (6 mm full-width at half-maximum). Subsequently, intensity normalization was performed using proportional scaling to reduce individual variation. The mean values of the functional networks (i.e., SN, HIPP, DMN, and FPN) were extracted from each scan based on the masks created by ICA. The metabolic levels of these brain networks were then compared between baseline and 12-month follow-up and correlated with the clinical scores.

### Statistical analysis

Continuous variables are presented as the mean and standard error. Statistical analysis was performed and graphs of the results were prepared using GraphPad Prism 9.0 software (GraphPad Software, Inc., San Diego, CA). Differences in clinical measurements among time points were identified using one-way repeated measures analysis of variance (ANOVA), as well as paired *t-*tests. Changes between the DBS-off and -on states were examined using multiple paired *t*-tests. After regressing the repeated measurement effect using linear mixed-effects models, correlation analysis was used to reveal the functional connectivity or dynamic state window changes (on − off) with the clinical score improvement (follow-up − baseline) and metabolic level with the clinical scores. The threshold was set at *P* < 0.05. Imaging results were considered significant after correction for the false discovery rate.

## Results

### Clinical measurements

**Fig. 2:**
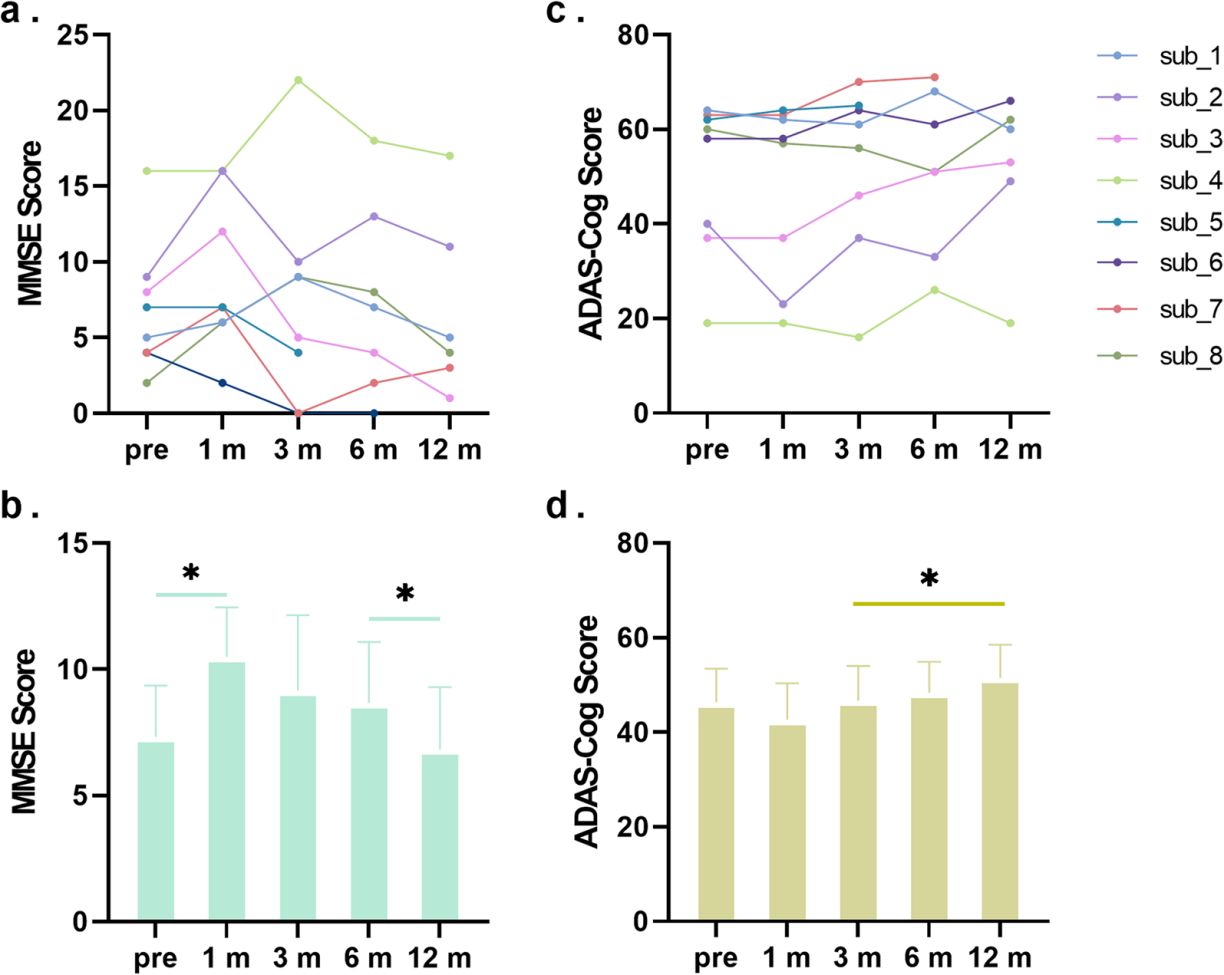
Changes to MMSE and ADAS-Cog scores over 12 months by treatment with NBM-DBS for all subjects. Improvements in MMSE scores are reflected by increased values (**a** and **b**), whereas improvements in ADAS-Cog scores are indicated by decreased values (**c** and **d**). Both individual (**a** and **c**, *n* = 8) and mean ± SE (**b** and **d**, *n* = 6) scores of the patients are shown. MMSE, Mini-Mental Status Examination; ADAS, Alzheimer’s Disease Assessment Scale. *P < 0.05 for paired *t*-test

DBS implantation was successfully completed for all eight patients (mean age, 66.6 ± 5.8 years). The surgery was well-tolerated by all patients and none developed adverse neurological effects. The demographic data, medication, target coordinates, and stimulation parameters are shown in Table 1. During the follow-up period, the clinical data of 6 patients (subjects 1, 2, 3, 4, 6, and 8) were collected at all time points. Two subjects failed to return to the hospital due to travel restrictions during the COVID-19 pandemic (subject 5, lacking data at 6 and 12 months; subject 7, lacking data at 12 months). The detailed clinical assessment scores of all eight patients are shown in Fig. 2a and c.

The average MMSE scores of the six patients who attended all follow-up examinations at all time points (i.e., baseline and 1, 3, 6, and 12 months) were 7.3 ± 5.0, 10.5 ± 4.8, 9.2 ± 7.3, 8.6 ± 5.9, and 6.8 ± 6.0, respectively. There was no significant difference in the average MMSE score among the time points (*P* > 0.05, one-way repeated measures ANOVA). After 12 months, the MMSE score, as compared to baseline, improved by > 10% in two patients, decreased by > 10% in two patients, and remained unchanged in two patients. Notably, at the 1-month point (3–5 days after stimulation), among the eight patients, the MMSE score improved in five patients, remained unchanged in two, and decreased in one (Fig. 2a). Further comparison between any two time-points revealed improvement only at the 1-month follow-up compared to baseline, and worse performance at 12 months as compared to that at 6 months (*P* < 0.05 for paired *t*-test, Fig. 2b).

The mean ADAS-Cog scores at baseline and at 1, 3, 6, and 12 months were 46.3 ± 17.4, 42.7 ± 19.0, 46.7 ± 18.0, 48.3 ± 16.1, and 51.5 ± 17.0, respectively. There was no significant difference among the time points (*P* > 0.05, one-way repeated measures ANOVA). After 12 months, the ADAS-Cog scores, as compared to baseline, was decreased by > 10% in two patients and remained unchanged in the other four (Fig. 2d). At the 1-month follow-up, the ADAS-Cog score was improved in one patient and remained stable in the other seven (Fig. 2c). Comparison between any two points showed no difference in the ADAS-Cog score between the follow-up and the baseline, and worse performance at the 12-month follow-up as compared to the 3-month (*P* < 0.05 for paired *t*-test, Fig. 2d).

### Multi-network functional connectivity during NBM-DBS

**Fig. 3:**
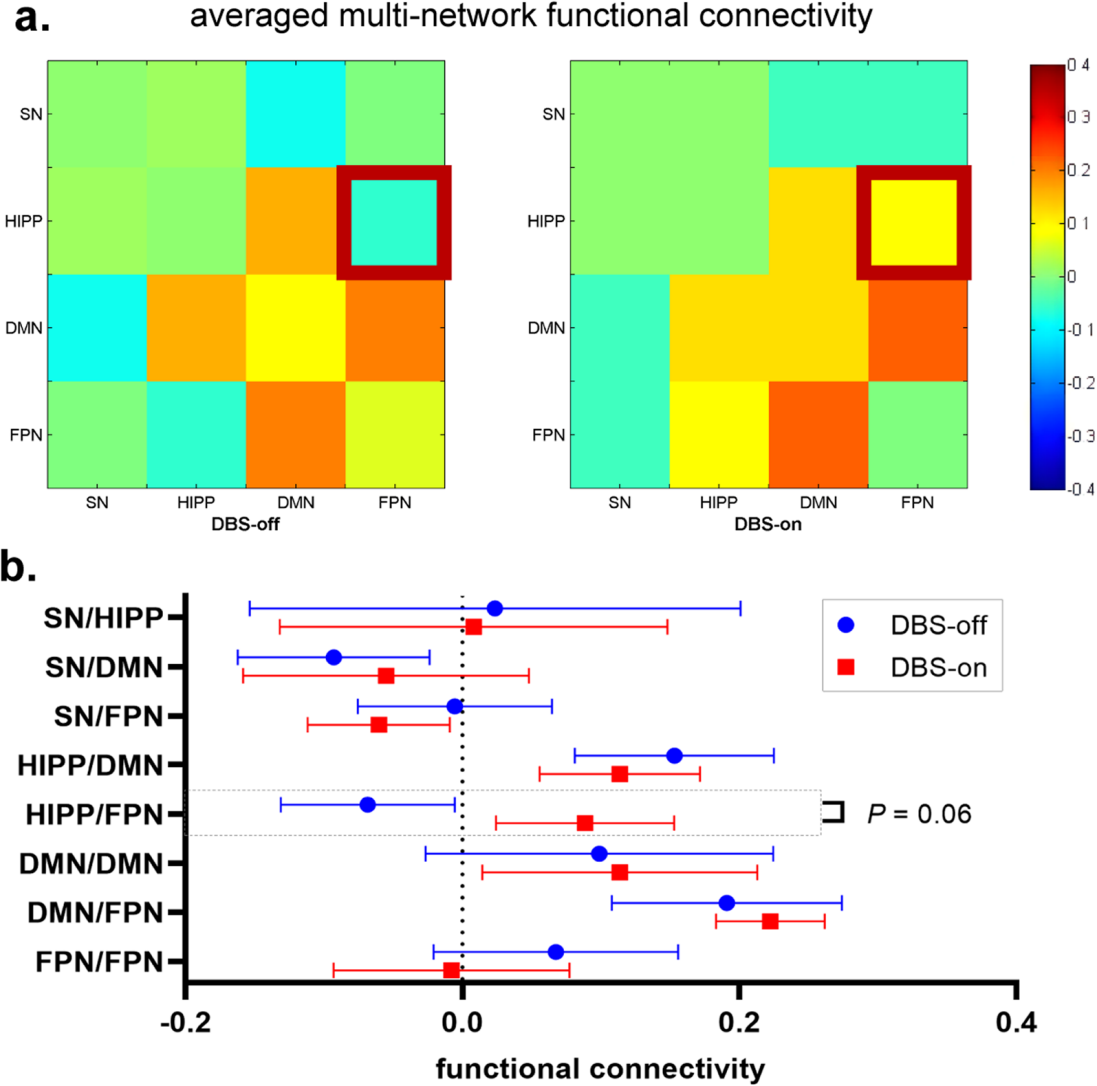
Multi-network functional connectivity modulated by NBM-DBS. **a** Averaged multi-network functional connectivity in the DBS-off and -on states. **b** Functional connectivity values were compared between the DBS-off and -on states within each connection. Data are presented as the mean ± SE (*n* = 6)

In the present study, fMRI data were successfully collected from six patients (subjects 1, 2, 3, 4, 5, and 6) at the 1-month follow-up, three patients (subjects 1, 2, and 4) at the 6-month follow-up, and three patients (subjects 2, 4, and 6) at the 12-month follow-up. Six components of the four resting-state networks (i.e., SN, HIPP, DMN, and FPN) were identified by ICA (Additional file 1: Fig. S1). Because improved MMSE performance was observed in these patients at the 1-month follow-up and the fMRI data in the DBS-off and -on states were successfully collected from six (75%) of the eight patients, the fMRI data in the DBS-off and -on states at the 1-month follow-up were compared. Although there was no significant difference in multi-network functional connectivity between the two states, functional connectivity between the HIPP and the FPN in the DBS-on state tended to increase (*P* = 0.06 without correction, Fig. 3).

### Seed-based functional network changes induced by NBM-DBS

**Fig. 4:**
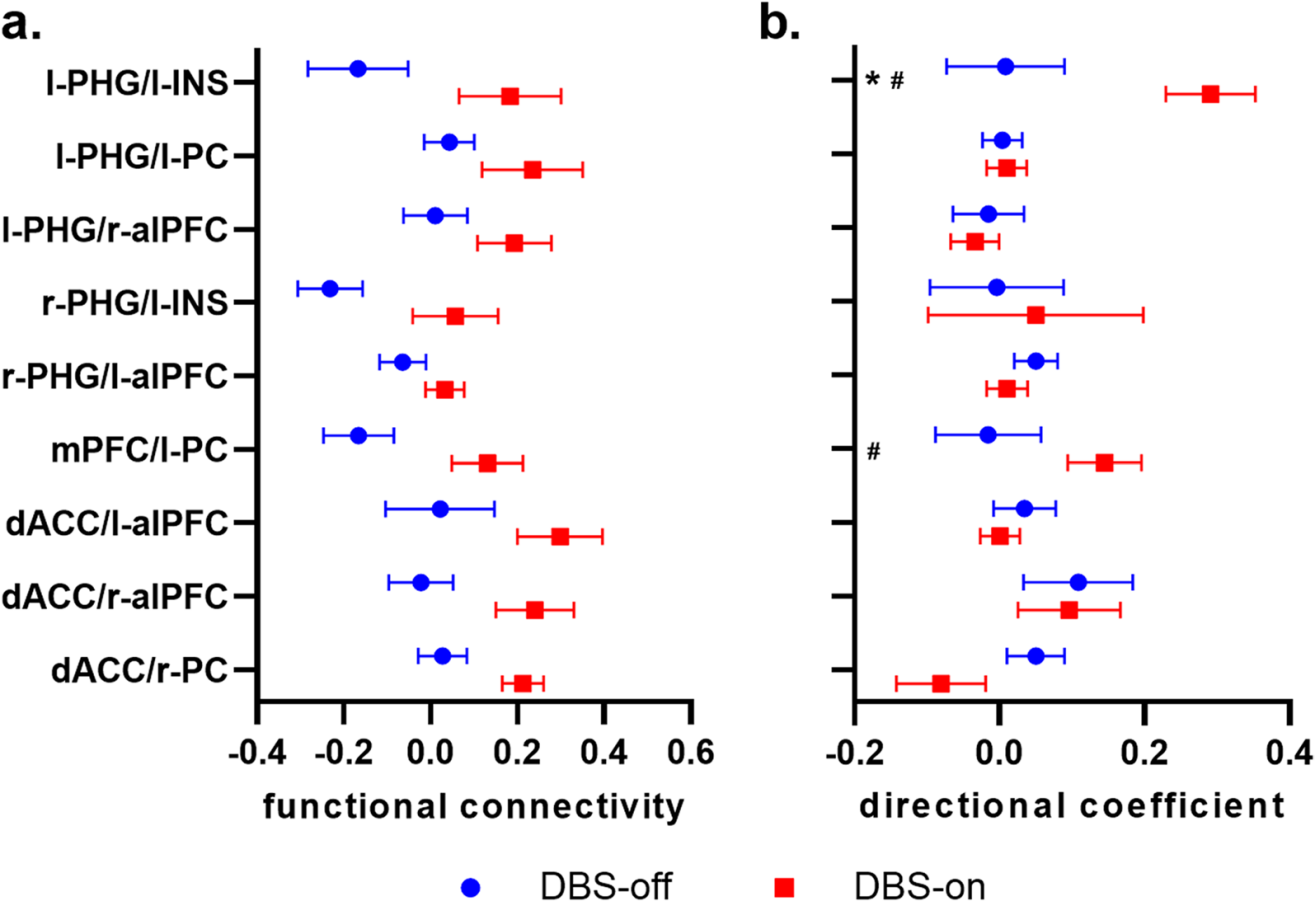
Functional connectivity among brain regions. **a** Functional connectivity values were modulated by NBM-DBS, and all these connections achieve *P* < 0.05 without correction of the false discovery rate. **b** NBM-DBS induced directional changes to the functional connections. Data are presented as the mean ± SE (*n* = 6). *DBS-off versus -on state, *P* < 0.05; ^#^compared with 0, *P* < 0.05

Seed-based functional connectivity was investigated among 14 regions in the four networks. The locations of the seeds were based on the highest *t*-value clusters determined by the one-sample *t*-test of the ICA maps. As compared to the DBS-off state, the functional connectivity of l-PHG/l-INS, l-PHG/l-PC, l-PHG/l-alPFC, r-PHG/l-INS, r-PHG/l-alPFC, mPFC/l-PC, dACC/l-alPFC, dACC/ r-alPFC, and dACC/r-PC showed a trend of increase (all *P* < 0.05 without correction of the false discovery rate) at DBS-on state at 1 month (Fig. 4a). Furthermore, GCA was performed on the causal influence flows of the changed connections. In the DBS-on state, l-PHG exerted a significant influence on l-INS, while mPFC exerted an influence on l-PC. Moreover, the directional coefficient of the l-PHG/l-INS connection significantly increased in the DBS-on state as compared to the DBS-off state (Fig. 4b).

Correlation analysis was also performed between the increased functional connectivity with clinical improvement (changes of the MMSE and ADAS-Cog scores). All 12 data points (including 6 at the 1-month, 3 at the 6-month and 3 at the 12-month) were included to increase the sample size. Following the use of a linear mixed-effects model to correct for repeated measurements, a negative correlation between the changes of l-PHG/l-PC connectivity and ADAS-Cog score ([on–off]/off) was observed (*r* = -0.89, *P* < 0.01).

### Dynamic functional connectivity changes during NBM-DBS

**Fig. 5:**
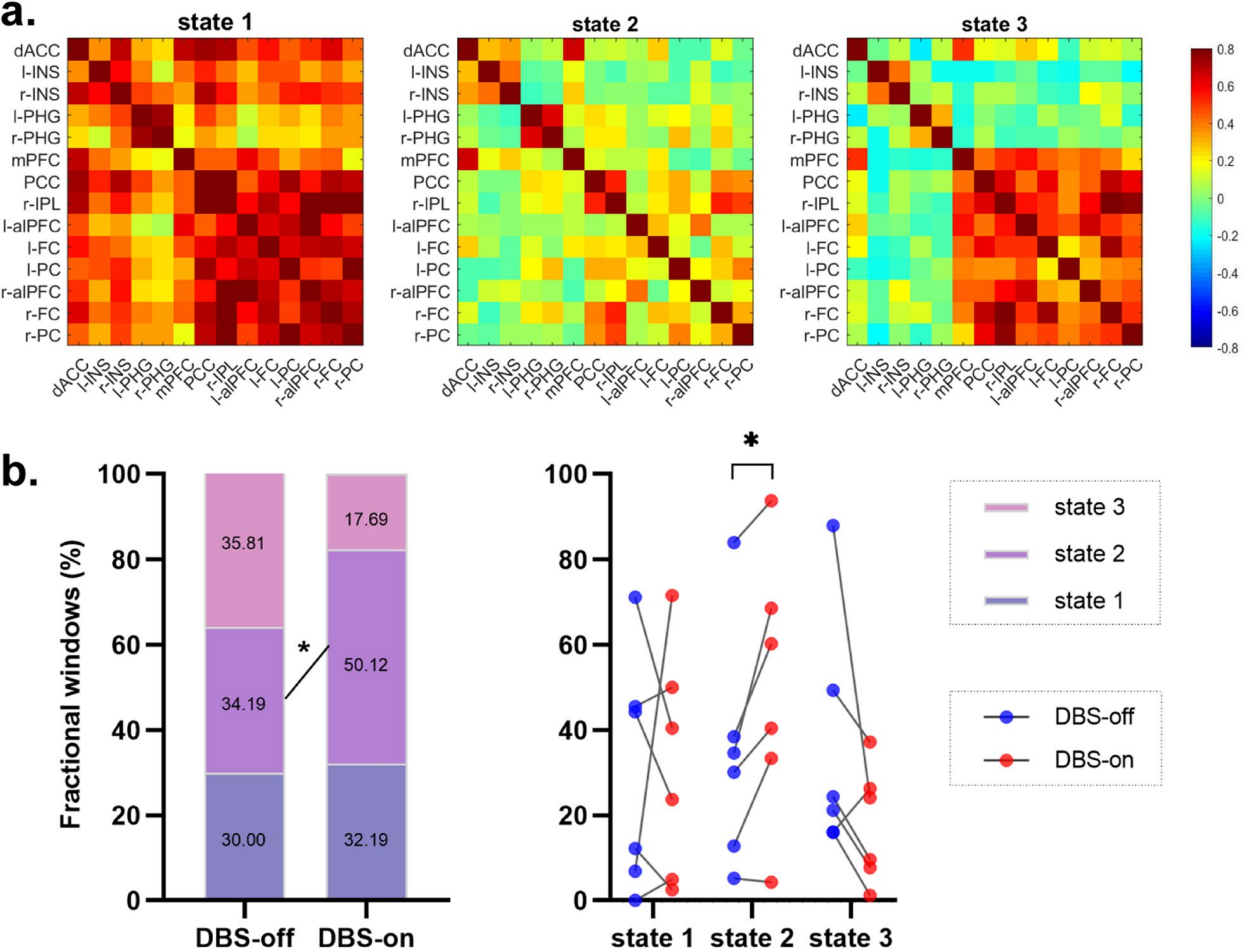
Dynamic functional connectivity changes by NBM-DBS. **a** Cluster centroids for each state. **b** Percentage of the total time of each DBS-off and -on state. The mean (left) and individual (right) data at 1 month are presented (*n* = 6), *P < 0.05

Three patterns of state of structured functional connectivity were identified, which recurred during individual scans and across subjects. State 1 showed a more frequent and stronger connection, state 2 showed relatively sparse connections, and state 3 showed specific strong connections between the DMN and FPN while other regions were less connected (Fig. 5a). At the 1-month follow-up, the three states occurred in similar proportions (fractional windows) in the DBS-off state (state 1, 30.00%; state 2, 34.19%; state 3, 35.81%). Compared to the DBS-off state, the proportion of state 2 was significantly increased (50.12%, *P* < 0.05), the proportion of state 3 tended to decrease (17.69%, *P* = 0.12) and the proportion of state 1 remained stable during the DBS-on state (32.19%) (Fig. 5b).

Correlation analysis of the changes of the proportion of state 2 with clinical improvement (rate of change of the MMSE and ADAS-Cog scores, on–off/off) was also performed. All 12 data points were included to increase the sample size. Following the use of a linear mixed-effects model to correct for repeated measurements, we found positive correlations between the proportion changes and the rate of change of the MMSE score (*r* = 0.72, *P* = 0.07), and negative correlations between the proportion changes and the rate of change of the ADAS-Cog score (*r* = -0.82, *P* < 0.05).

### PET imaging outcomes

**Fig. 6:**
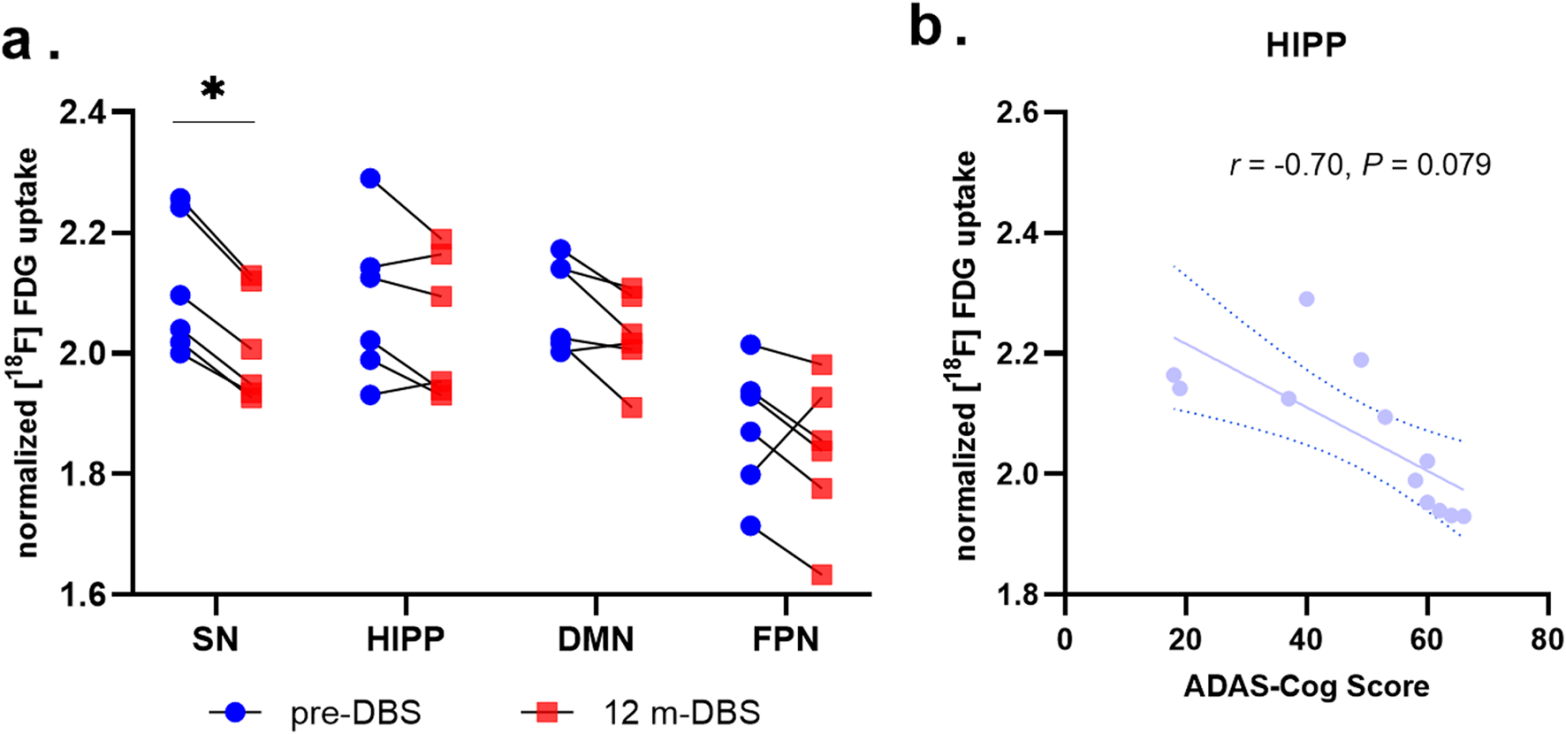
Cerebral glucose utilization (normalized to the whole brain) of all patients as determined by PET. **a** Normalized [^18^F] FDG uptake in different networks before and 12 months after NBM-DBS. Data are presented as the mean ± SE (*n* = 6), *DBS-off versus -on state, *P* < 0.05. **b** Correlation between cerebral glucose utilization in the HIPP and ADAS-Cog score, both pre-DBS and at 12 months (12 dots)

Six patients underwent PET scans both at baseline and at 12-month follow-up (subjects 1, 2, 3, 4, 6, and 8). ^18−F^FDG uptake decreased significantly by 4.66% in the SN at the 12-month follow-up, and no significant decrease was observed in the HIPP, DMN, and FPN (decreased by 1.83%, 2.62%, and 2.25%, respectively) (Fig. 6a). In addition, following the use of a linear mixed-effects model to correct for repeated measurements, a trend of negative correlation was observed between ^18−F^FDG uptake in the HIPP and the ADAS-Cog score (*r* = -0.70, *P* = 0.079) (Fig. 6b).

## Discussion

In this study, eight patients with moderate-to-severe AD underwent NBM-DBS. At the 12-month follow-up, both clinical assessment and neuroimaging (fMRI and PET) were performed. The main findings were: (1) there was a temporary improvement in the MMSE score at the 1-month follow-up; (2) functional connectivity between the HIPP and FPN tended to increase during the DBS-on state, with a negative correlation between the DBS-induced change of the l-HIPP/l-PC functional connectivity and the rate of change of the ADAS-Cog score; and (3) changes of proportions of different states of dynamic functional connectivity during the DBS-on state were correlated to clinical improvement.

In this study, low-frequency (20 Hz) stimulation was employed based on previous basic and clinical studies of NBM-DBS [5, 26, 27], because it has potentially excitatory effect on NBM neurons and stimulates acetylcholine release from the cortical terminals of fibers originating in the NBM [28]. However, there was no difference in cognitive function between the baseline and the 12-month follow-up, consistent with findings of previous double-blind clinical studies [5, 26, 27, 29]. Interestingly, positive clinical efficacy was observed in the early stage of treatment. At the 1-month follow-up, the MMSE scores of five patients and the ADAS-Cog score of one patient were improved. In addition, the efficacy of continuous stimulation varied at the 3-month follow-up, with MMSE scores being decreased in four patients while ADAS-Cog scores being increased in three patients. Similarly, several animal studies reporting memory enhancement induced by NBM-DBS with relatively shorter stimulation periods (≤ 100 days) [30–32] and others indicated that the timing of DBS is key to the effect [33]. In addition, intermittent NBM-DBS, as compared to continuous stimulation, resulted in better cognitive performance in both rodents and primates [34, 35]. These findings suggested that although NBM-DBS is a potential treatment for dementia, the commonly used stimulation parameters may not be optimal. In future DBS studies in AD, individualized intermittent stimulation and closed-loop stimulation could help to improve the long-term efficacy [36].

Furthermore, the PET data revealed a slight decrease of FDG uptake in the HIPP, DMN and FPN by 1.83%–2.62%, and a significant decrease in SN by 4.66%. Since AD is a progressive disease, untreated AD patients show an average decrease of FDG uptake by 5.2% per year versus 0.9% in healthy controls [37]. Our findings  imply that NBM-DBS may slow down the reduction of glucose metabolic rate in multiple brain areas. Similar results have also been found in previous studies on mild-to-moderate AD patients [1, 5]. In addition, we found a trend of correlation between the metabolic level of the HIPP and cognitive performance, which further indicates that PET-FDG in specific brain areas could reflect changes in dementia status.

To better understand how NBM-DBS modulates the brain networks in AD patients, resting-state functional connectivity was compared between the DBS-off and -on states. After ICA, four networks (SN, HIPP, DMN, and FPN) were selected for further analysis because they have been reported to be structurally and functionally abnormal in AD patients [38–41]. Disruption of local functional networks manifested as low clustering coefficients and decreased degree of network connection, is associated with high levels of amyloid plaques and tau tangles in multiple cortical regions of the frontal, insular, limbic, temporal, and parietal lobes [39]. The SN-DMN-FPN cross-network functional connectivity is also reported to be significantly impaired in AD patients [41]. The NBM is connected to the entire neocortex [42] and the integrity of the related white matter projections is strongly associated with cognitive degeneration [43]. Additionally, the cortical thickness of the frontal, parietal, and temporal lobes is reported to be associated with the clinical outcome of NBM-DBS [44]. Thus, NBM-DBS is expected to be a promising approach to treating dementia by enhancing the activities of multiple cortical areas and connectivity in large-scale brain networks. However, relatively few studies have investigated the modulatory effect of NBM-DBS on brain networks. Previous studies in patients with Parkinson’s disease dementia (*n* = 6) and dementia with Lewy bodies (*n* = 4) found that NBM-DBS did not significantly alter the regional functional connectivity [26, 27]. The possible explanations for this finding are: (1) a small sample size; (2) the related clinical efficacy was not significant; and (3) limitation of functional connectivity analysis to the seed-to-voxel level and determination of seed location according to previous studies. In the present study, the comparison between the DBS-off and -on states was mainly focused on the 1-month follow-up, at which point the MMSE score was significantly improved. Moreover, although the location of each seed was determined based on the one-sample *t*-test of the ICA component maps, inter-network comparisons indicated that functional connectivity between the HIPP and FPN during NBM-DBS tended to increase. Further seed-to-seed analysis showed increased functional connectivity of PHG/alPFC and PHG/PC. Besides the HIPP and FPN, functional connectivity of SN/FPN, SN/HIPP, and DMN/FPN at the seed-to-seed level during the DBS-on state also tended to increase, and causal influence flows from l-PHG to l-INS and mPFC to l-PC were also observed. These findings suggested that NBM-DBS modulates the functional connectivity of multiple networks in AD patients.

The results of the present study also showed that the HIPP was key to the neuromodulation by NBM-DBS in AD patients. It is generally accepted that the hippocampus is critical to memory, navigation, and cognition [45, 46]. Pathologic changes of the structure and function of the hippocampus occur in the early stage of AD and the severity of such impairments is reportedly associated with the extent of cognitive decline [47–50]. Similarly, our PET data revealed a correlation between the metabolic level of the HIPP and cognitive performance. In addition, in patients with dementia, a delta/theta band network between the NBM and PHG was detected [51]. Cholinergic degeneration in the NBM could damage cortical projections specifically to the PHG and temporal lobe, leading to cognitive deterioration [52]. Thus, it is reasonable to speculate that the HIPP is a key modulator of NBM-DBS. In the present study, as compared to the DBS-off state, the connectivity between the HIPP and FPN tended to increase during the DBS-on state, and changes of the PHG/PC connectivity were negatively correlated to the rate of change of the ADAS-Cog score, which is positively correlated to clinical improvement. Based on the seed-to-seed analysis, the PHG/INS and PHG/alPFC connectivity tended to improve. Furthermore, dynamic functional network analysis demonstrated that NBM-DBS significantly increased the proportion of state 2, while the proportion of state 3 tended to decrease. As compared to state 3, connectivity in state 2 between PGH and regions in other networks (especially the DMN and FPN) was relatively increased. State 3 was characterized by strong connections specifically between DMN and FPN, while other regions were less connected. Similarly, increased FPN connectivity in AD patients has also been reported in previous studies [53, 54]. In addition, the percent change of state 2 was associated with clinical improvement, further suggesting the important role of the HIPP in cognitive regulation by NBM-DBS. It needs to be mentioned that the functional connectivity of both DBS-on and -off states was measured on the same day of follow-up, whereas the cognitive performance change was calculated at different time points. Thus, the correlation results may reflect the sensibility of functional connectivity to the relatively long-time efficacy other than the immediate treatment effect of DBS. Moreover, although a correlation was revealed between the connectivity change and cognitive improvement, if the ADAS-Cog score comparison achieves significance in group analysis, the finding would be more convincing.

Similar to previous reports [55, 56], only a relatively small number of AD patients met the inclusion criteria and even fewer agreed to undergo surgery. In addition, patients with severe AD often cannot adhere to the protocol of fMRI when they are awake. Thus, the main limitations of the present study were the small sample size and incomplete follow-up, limiting the significance of the results. Also, the NBM was not included in the network analysis because MRI would be affected by magnetic field artifacts after surgery. Thus, the results of the present study can only offer evidence of changes of other brain networks during NBM-DBS. Lastly, continuous low-frequency NBM-DBS did not achieve long-term efficacy. Hence, future studies are warranted to investigate intermittent stimulation, phase-locking of gamma oscillation modulation, and closed-loop stimulation, as mentioned in recent studies [35, 57], to achieve better clinical outcomes.

## Conclusion

The results of the present study indicated that NBM-DBS can improve cognitive performance in patients with advanced AD in a short time, which may be related to the modulation of the connectivity patterns of multiple networks, in part via the hippocampus. Overall, these findings show evidence of brain network response to NBM-DBS in patients with advanced AD and provide a mechanism underlying network modulation, which may facilitate clinical adjustment of new stimulation patterns.

## Supplementary Information

**Additional file 1. Fig. S1.** Resting-state network components selected in AD patients. **Fig. S2.** Selected seeds in the different resting-state networks.

## Data Availability

The datasets used and/or analysed during the current study are available from the corresponding author on reasonable request.

## Abbreviations

AD: Alzheimer's Disease
DBS: Deep brain stimulation
NBM: Nucleus basalis of Meynert
PET: Positron emission tomography
MRI: Magnetic resonance imaging
MMSE: Mini-Mental State Examination
ADAS-Cog: Alzheimer’s Disease Assessment Scale
fMRI: Functional MRI
ICA: Independent component analysis
SN: Salience network
HIPP: Hippocampal network
DMN: Default mode network
FPN: Frontoparietal network
dACC: Dorsal anterior cingulate cortex
INS: Insula
PHG: Parahippocampal gyrus
mPFC: Medial prefrontal cortex
alPFC: Lateral prefrontal cortex
GCA: Granger causality analysis
SUV: Standardized uptake value
