# Supplementary material for: Deep brain stimulation of the nucleus basalis of Meynert modulates hippocampal–frontoparietal networks in patients with advanced Alzheimer’s disease
Source: Transl Neurodegener. 2022 Dec 5;11:51. doi: 10.1186/s40035-022-00327-9 (PMC9721033; doi:10.1186/s40035-022-00327-9)
Supplement: Supplementary file 1 — Additional file 1. Fig. S1. Resting-state network components selected in AD patients. Fig. S2. Selected seeds in the different resting-state networks. [file 40035_2022_327_MOESM1_ESM.docx]

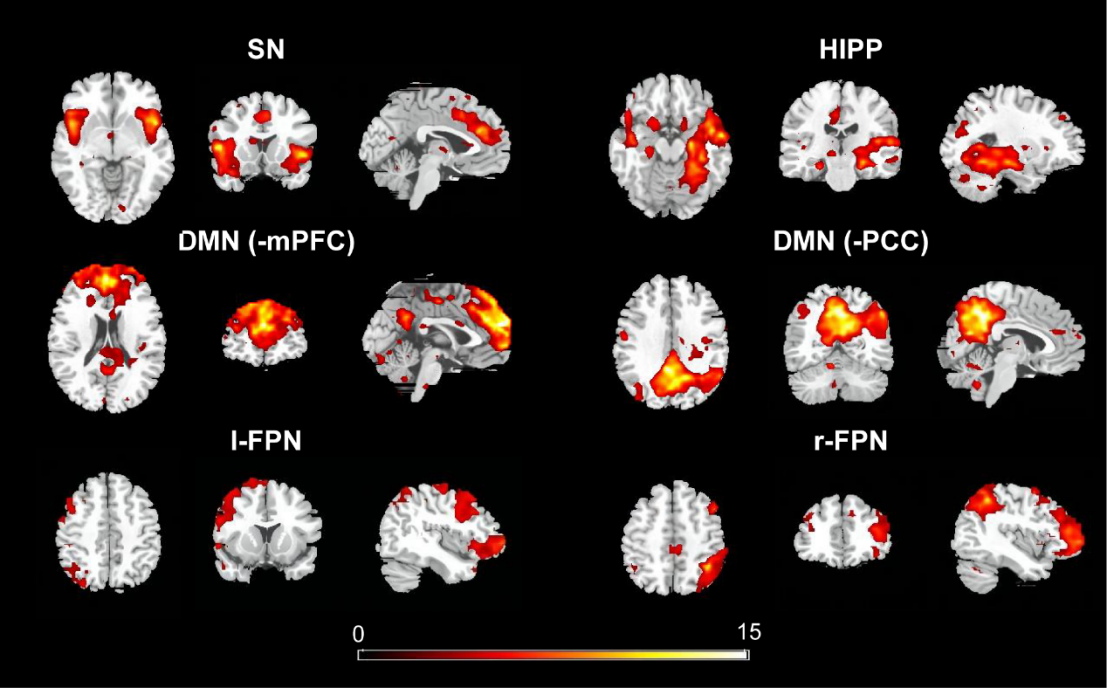


**Figure S1.** Resting-state network components selected in AD patients. SN, salience network; HIPP, hippocampal network; DMN, default mode network; FPN, frontoparietal network.


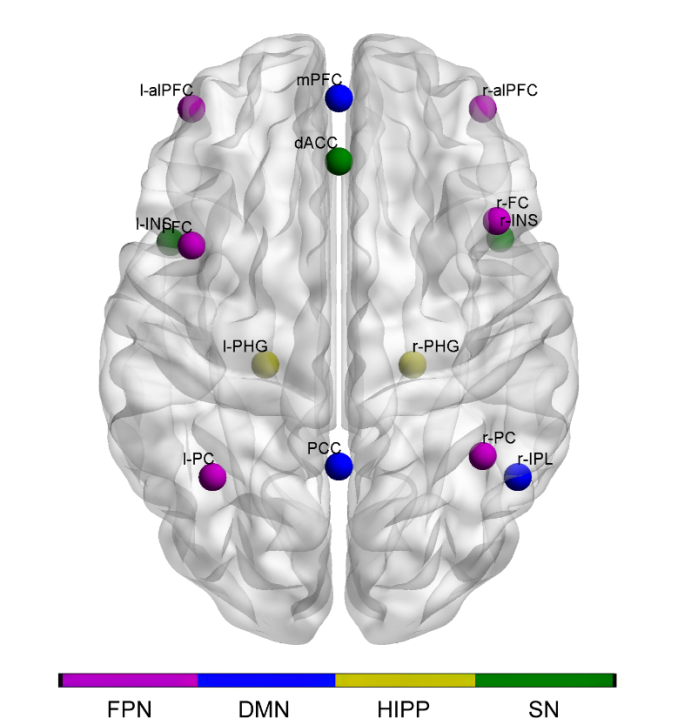


**Figure S2.** Selected seeds in the different resting-state networks.
